# Molecular docking analysis of the BRCA1 protein with compounds from Justica adhatoda L

**DOI:** 10.6026/97320630016888

**Published:** 2020-11-30

**Authors:** Selvaraj Jayaraman, Vishnupriya Veeraraghavan, Radhika Nalinakumari Sreekandan, Surapaneni Krishna Mohan, Sumetha Suga Deiva Suga, Devakumar Kamaraj, Sonaimuthu Mohandoss, Sravanthi Koora

**Affiliations:** 1 Department of Biochemistry, Saveetha Dental College and Hospitals, Saveetha Institute of Medical and Technical Sciences, Saveetha University, Chennai - 600 077, India; 2 Department of Clinical Skills & Simulation, Panimalar Medical College Hospital & Research Institute, Varadharajapuram, Poonamallee, Chennai - 600 123; 3 Department of Biochemistry and Department of Clinical Skills & Simulation, Panimalar Medical College Hospital & Research Institute, Varadharajapuram, Poonamallee, Chennai - 600 123; 4 Department of Microbiology, Panimalar Medical College Hospital & Research Institute, Varadharajapuram, Poonamallee, Chennai - 600 123; 5 Department of Pharmacology, Panimalar Medical College Hospital & Research Institute, Varadharajapuram, Poonamallee, Chennai - 600 123; 6 School of Chemical Engineering, Yeungnam University, Gyeongsan, Gyeongbuk-do, 38541, Republic of Korea; 7 Department of Pharmacology, Government Medical College Siddipet, Siddipet-502 103, Telangana, India

**Keywords:** BRCA1, Justica adhatoda L, Molecular docking

## Abstract

BRCA1 is a human tumour suppression gene. Therefore, it is of interest to document the Molecular docking analysis data of the BRCA1 protein with compounds from Justica adhatoda L (adhatoda). We report that Amrinone, Hexadecanoic acid, Pyrazinamide & Vasicinone have acceptable binding features with the BRCA1 protein for further consideration.

## Background

Breast cancer is the most prevalent form of cancer diagnosed and the second largest cause of death between women in Western countries [1]. The vast majority of breast cancers become sporadic, however up to 10% may be due to hereditary predisposition. Of these hereditary cancers, a significant part is due to mutations in the germ line within the BRCA1 gene [2]. Moreover, the expression of functional BRCA1 protein has been lost to a significant proportion of sporadic breast cancers, indicating that such a mechanism plays a significant role in breast carcinogenesis [3]. Different BRCA1 functions can lead to its tumour suppression activity; include DNA repair roles, cell cycle control and transcriptional regulation [4]. BRCA1 was assigned to chromosome 17q21 [5]. It contains a nuclear protein of 1,863 amino acids that regulates, at least in part, transcriptional activation, DNA repair, apoptosis, cell cycle regulation, and chromosomal remodeling [6]. BRCA1 is a classical tumour suppressor gene for family breast cancer [7]. The presence of hereditary mutations in BRCA1 appears to become one of the most well defined overall risk factors for breast cancer development; moreover, such family mutations, along with family BRCA2 mutations, appear in less than 10% of all cases diagnosed [8]. By definition, BRCA1 gene mutations in germ line seem to be practically invisible in sporadic breast cancers [9]. Studies of BRCA1 functions in DNA repair and genome consistency had also emerged in the field of BRCA1 that since discovery. In comparison, identifying the intriguing tissue-and gender-specificity linked with BRCA1 tumour suppression is a less travelled route. While the role of oestrogen with in growth of sporadic breast cancer has indeed been extensively studied, the possible interaction among BRCA1 and hormone synthesis and actions in the field of BRCA1 has been strangely under-appreciated and under-investigated. However, the remarkable restriction of BRCA1-related tumours to large hormone-responsive tissues begs the question on whether the tumour suppressor role of BRCA1 is connected to a hormone homeostasis throughout the breast and ovaries. In addition, epidemiological data have shown that prophylactic oophorectomy in women with BRCA1 mutations reduces the incidence and recurrence of breast cancer by 75% [10-12]. Therefore, it is of interest to document the molecular docking analysis data of the BRCA1 protein with compounds from Justica adhatoda L (adhatoda).

## Materials and Methods:

### Protein Preparation:

The target protein structure of BRAC1 has been retrieved from the Protein Data Bank (PDB ID: 1T15). The input file was created by removing water molecules, ions, ligands and subunits from the original file. Kollman charges and polar hydrogen atoms are applied to the PDB receptor file for the preparation of the receptor protein for docking simulation.

### Ligand Preparation:

The 12- compounds of Justica adhatoda L (Table 1 - see PDF) has been retrieved from the PubChem compound database. It had been prepared with the ChemBioDraw and the MOL SDF format of this ligand has been translated to a PDBQT file using the PyRx method to produce atomic coordinates.

### Molecular Docking:

The computational ligand-target docking technique was used to evaluate the structural complexes of BRAC1 (target) with Justica adhatoda L compounds (ligand) in order to recognize the structural basis of this target protein specificity. Finally, the docking was performed using PyRx, the AutoDock Vina option based on the score feature. "grid point" is applied to the energy of interaction of Justica adhatoda L compounds with BRAC1. At each stage of the simulation, ligand and protein interaction energy was evaluated using grid-based atomic affinity potentials. The remaining parameters have been set as usual.

### Molecular Docking Visualization:

Ligand protein complexes have been visualized with PyMol and Discovery Studio Visualizer to see the model interactions between the compounds and indeed the target amino acid protein residues.

## Results and Discussion:

**Figure 1:**
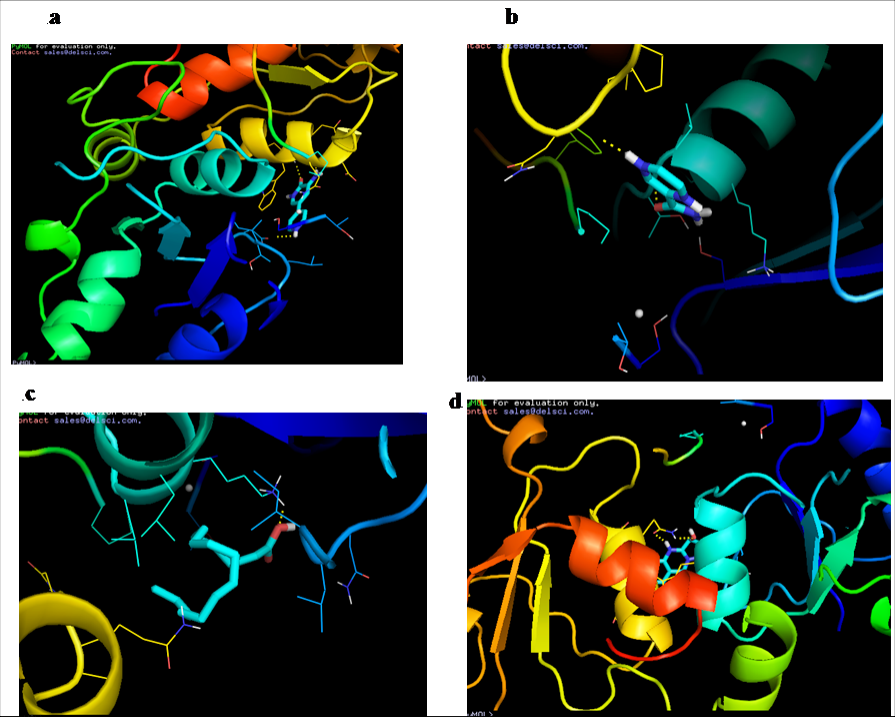
Molecular interaction BRAC1 protein with a) Amrinone, b) Pyrazinamide, c) Hexadecanoic acid and d) Vasicinone.

High throughput Virtual Screening (HTVS) programmes including such PyRx with graphical user interfaces (GUIs) that are used to predict receptor-ligand interactions were helpful for ligand comparison studies as they provide well-integrated storage and visualisation of HTVS results that facilitate binding analysis. AutoDock 4.0 module (present in PyRx 0.8 Python prescription 0.8 package) was utilized for molecular docking studies. During the silicon docking process, conformers have been rated as per their estimated free binding energy. The top docked solutions have been rated on the basis of energy scores, as the docking software and score feature are good at removing compounds that would not match well with the active site electrically or sterically. A reduced free binding energy suggests a much more reliable protein-ligand relationship and a higher affinity between protein and ligand. The conformation with minimum energy binding value suggested the better interaction posing. Table 2(see PDF) revealed the energy binding values of Justica adhatoda L compounds. Depending on the binding energy, the best four compounds have been identified and their interaction with BRAC1 protein has been analysed using the Pymol visualization method. Figure 1a provided the mode of interaction between ligands in the binding pocket of the target protein. Figure 1a demonstrates how Amrinone interacts with BRAC1 protein. The three hydrogen bond interactions with BRAC 1 protein were formed by ILE-680, GLN-1785, & ASP-1778 amino acids. The docked pose of BRAC1 with Pyrazinamide has been seen in Figure 1b and Hexadecanoic acid & Vasicinone has been shown in Figure 1c &Figure 1d actually indicating the binding position of the ligand to the protein. As shown in Table 2 (see PDF), Pyrazinamide displayed binding energy-5.2 kcal / mol which also had higher binding energy compared to Hexadecanoic acid (-4.9 kcal / mol) and Vasicinone (-4.4 kcal / mol). In addition, Pyrazinamide formed two hydrogen bond interactions through amino acid, namely LEU-1701 & ASN-1774, while Hexadecanoic acid & Vasicinone had one interaction through LYS-1702 & GLN-1779, respectively.All ligands have been successfully docked and placed in a binding pocket of targets in the same region. Even so, the method of interaction between ligands and molecular targets needs to be explored in experimental analysis.

## Conclusion

We report the molecular binding features Amrinone, Hexadecanoic acid, Pyrazinamide & Vasicinone with the BRCA1 protein for further consideration.
